# A novel universal primer pair for prokaryotes with improved performances for anammox containing communities

**DOI:** 10.1038/s41598-020-72577-4

**Published:** 2020-09-24

**Authors:** Lorenzo Mazzoli, Giulio Munz, Tommaso Lotti, Matteo Ramazzotti

**Affiliations:** 1 Department of Experimental and Clinical Biomedical Sciences “Mario Serio”, University of Florence, Florence, Italy; 2 Department of Civil and Environmental Engineering, University of Florence, Florence, Italy

**Keywords:** Classification and taxonomy, Databases, High-throughput screening, Biogeochemistry, Environmental sciences, Bioinformatics

## Abstract

Abundance profiling via 16S rRNA targeted next generation sequencing (NGS) is a common procedure to characterize mixtures of prokaryotic populations inhabiting an environment. Depending on the variable region/s addressed, different maps can be obtained due to their different information content. In this work, we focussed on wastewater microbial communities and we compared several recently developed universal primers that addressed regions V1-V3, V3-V4 and V4. They all proved to have good performance over a wide range of microbial phyla, but the phylum Planctomycetes was not optimally covered, especially for members of the Brocadiales family. Such bacteria are at the basis of the novel nitrogen removal strategy based on anammox process. To overcome this limitation we performed an extensive bioinformatic analysis that allowed the design of a primer (Pro341FB) that shows increased sensitivity for this specific phylum with respect to the previously proposed Pro341F primer. Upon validation using a 16S NGS survey on microbial communities from different wastewater treatment plant (activated sludge systems, anaerobic digesters, aerobic and anaerobic granules) we demonstrated that Pro341FB is able to reveal up to 5 times more members of the Candidatus Brocadiales family (plus many other previously under-covered prokaryotes) than Pro341F, without affecting its excellent previous coverage.

## Introduction

The autotrophic nitrogen removal process is an increasingly applied biological process for nitrogen removal and is regarded as the most economical process, at present, for the treatment of N-rich wastewaters^1^. The process is based on two distinct autotrophic biological processes in cascade: the aerobic conversion of about half of the ammonium to nitrite (Partial Nitritation, PN) and the formation of molecular nitrogen via the oxidation of the remaining ammonium, using the formed nitrite as electron acceptor (ANaerobic AMMonium OXidation, anammox). Besides the common Ammonia Oxidizing Bacteria (AOB) catalyzing PN, the process requires the presence of a relatively novel bacterial species belonging to the order “*Candidatus Brocadiales*” (phylum *Planctomycetes*) to catalyze the anammox process^2^. The Bergey's Manual of Systematic Bacteriology, that frames the RDP database and its classification system^3^, defines members of the anammox community as belonging to the full lineage kingdom *Bacteria*, phylum P*lanctomycetes*, class *Planctomycetia*, order “*Candidatus Brocadiales*”, family *Candidatus Brocadiaceae* (with 5 associated genera). Up to date, ten anammox species have been described, including seven that are available in laboratory enrichment cultures^4^. They all have the taxonomic status of *Candidatus*, as none can be maintained in a microbiological culture collection, namely cannot be cultured as classical pure cultures. Known species are divided into five genera: *Kuenenia*, *Brocadia, Anammoxoglobus, Jettenia* and *Scalindua*. Such microbes are able to oxidize ammonia using nitrite as electron acceptor and, in oder to remove nitrogen (usually present as ammonia in wastewater), a metabolic interaction with AOB is needed in order to remove nitrogen^5^. When specific selecting conditions are applied, this microbial consortium can grow within a highly organized nearly spherical biofilm structure called granule, composed and scaffolded by extracellular polymeric substances (EPS) mainly composed by exopolysaccharides and proteins^6^. Until now, the presence of *Brocadiales* has been measured and quantified by amplifying and (optionally) sequencing amplicons obtained using specific primers.

In order to understand the complexity of the metabolic activity within a granule, much more details are needed besides knowing the proportion and amount of *Brocadiales*. In fact, a granule presents a consortium of metabolically interacting species whose presence and activity is mainly controlled by oxygen availability, in a spatial (radial in this case) gradient. Accordingly, a main interest, in this case is to know the proportions of microbes in the whole granules community, especially for monitoring and managing the development of granules in a reactor (both in pilot studies or at a large scale).

To this aim, a number of primers pairs claimed to be universal have been developed during the years. Two recent surveys aimed at obtaining “universal” primers are particularly relevant for this work, namely the 2014 study by Takahashi et al.^7^ and the 2015 study by Albertsen et al.^8^. They both addressed the V3 and V4 hypervariable regions as the most useful and identified different primer pairs (reported in Table 1) supposed to be comprehensive for prokaryotes.

**Table 1:** sequences of the primers used in this study.

Name	Sequence (5′-3′)	Reference
Pro341FB	CCTACGGGNBGC**W**SCAG	This work
Pro341F	CCTACGGGNBGCASCAG	Takahashi et al.^7^
Pro805R	GACTACNVGGGTATCTAATCC	Takahashi et al.^7^
314F	CCTACGGGNGGCWGCAG	Albertsen et al.^8^
805R	GACTACHVGGGTATCTAATCC	Albertsen et al.^8^
27F	AGAGTTTGATCCTGGCTCAG	Albertsen et al.^8^
534R	ATTACCGCGGCTGCTGG	Albertsen et al.^8^
515F	GTGCCAGCMGCCGCGGTAA	Albertsen et al.^8^
806R	GGACTACHVGGGTWTCTAAT	Albertsen et al.^8^
Fwd MiSeq Adapter	TCGTCGGCAGCGTCAGATGTGTATAAGAGACAG	
Rev MiSeq Adapter	GTCTCGTGGGCTCGGAGATGTGTATAAGAGACAG	

The modification introduced to the Pro314F primer is indicated in bold in Pro314FB. The sequences of the MiSeq adapter used in this work for amplicon generation and subsequent sequencing are also reported.

The primer pair described Takahashi et al.^7^ (Pro341F/Pro805R) was reported to match approximately 98.0% of Bacteria and 94.6% of archaea rRNA gene sequences present in the RDP database, surpassing other primer pairs tested (noticeably 341F/R806 by Muyzer^9^ and Caporaso^10^) and proved good performances and limited bias on 16S libraries from pig fecal samples (in agreement with qPCR measurements).

The set of primers described in Albertsen et al.^8^ was interesting because they were tested applied on activated sludge communities. The results here indicate V4 as the optimal target region (targeting 95.5% of the considered phyla), but only slightly better than the V3-V4 that had a theoretical general coverage of around 95%.

In this work we demonstrate that such primers have severe limitations in appropriately quantifying *Brocadia* and we present the result of an in silico screening aimed at characterizing primers able to maximize the coverage of *Brocadiales* while maintaining intact performances in terms of taxonomic coverage than those detailed above. The improved fidelity of the proposed primer for the V3-V4 region of the bacterial/archaeal 16S gene was tested by NGS in several real wastewater treatment plant (WWTP) -associated microbial communities, including one composed by anammox granules.

## Results

### Estimation of a generalized wastewater treatment plant microbial community

In order to perform a deep taxonomic survey of microbial communities associated to wastewater treatment, we initially surveyed the EBI MGnify database^11^, collecting abundance profiles obtained by 16S amplicon based surveys of wastewater treatment communities.

We were able to roughly identify 1465 prokaryotic genera in 3433 samples from 49 studies (see Supplementary Data S1.1), with members of the archaea kingdom in about 22.5% of samples. When we restricted the analysis on sludges the number of studies was reduced to 33 with 1363 samples, however we identified 1379 genera and the number of samples showing archaea was about 40% (see supplementary data S1.2). Such observation underlined the relevance of archaea in wastewater environments. Interestingly, in the wastewater biome we found 128 samples (about 3.7%) from 24 studies (about 50%) showing evidence of anammox species, a percent that grew to about 5% in sludge samples from 10 studies (30%). This result manifests the need of properly taking into account anammox communities when estimating microbial abundance profiles in such environments.

### Evaluation of existing primers

We then sought to verify whether existing primer pairs with established high performances and good coverage over the widest range of microbial species were able to appropriately cover wastewater associated communities, especially for the anammox components, using the most updated 16S RDP collection.

**Figure 1:**
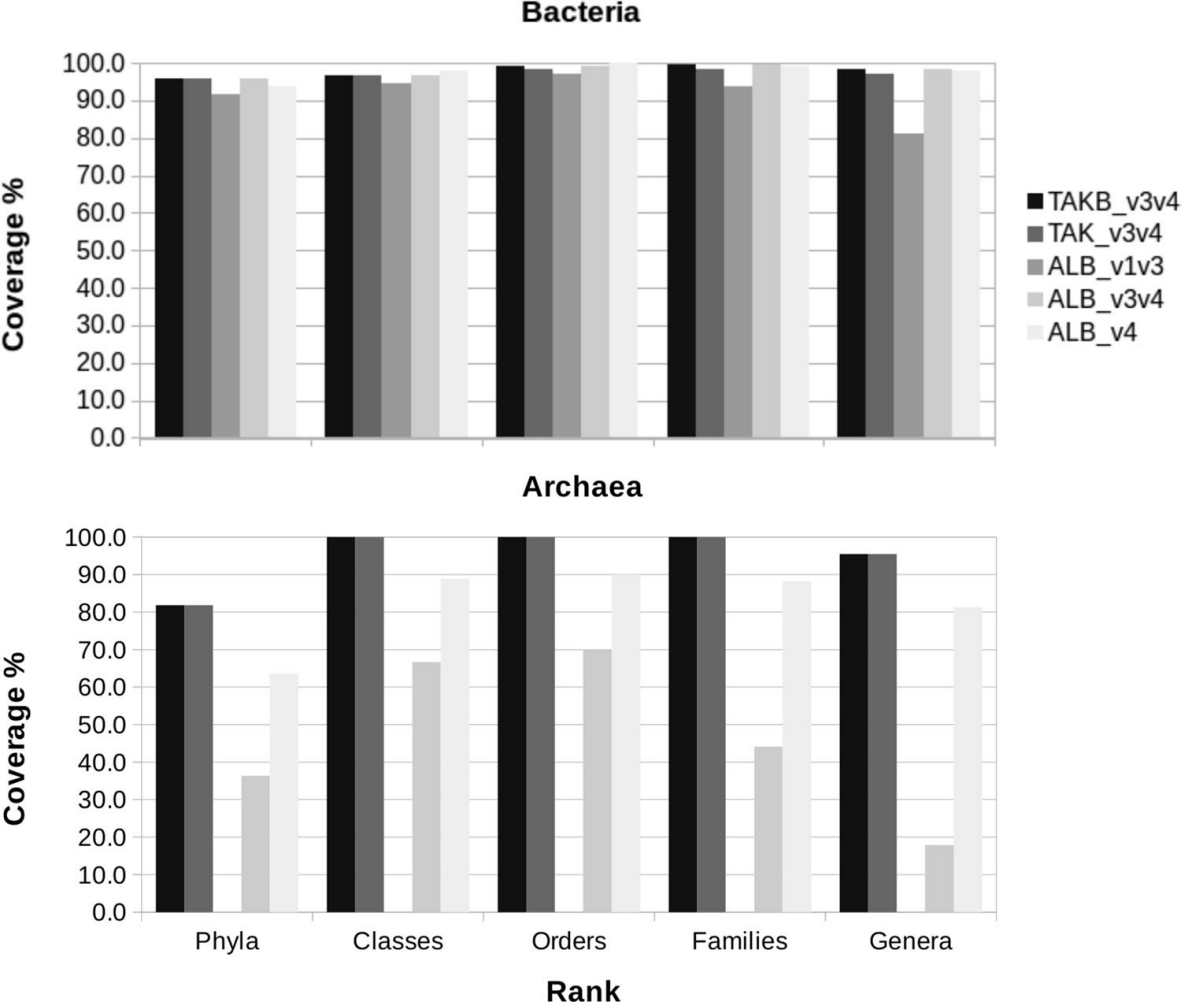
Comparison of the overall theoretical performance in coverage (percent of members of the given rank mapped) of the different primer pairs used in this study.

All Takahashi et al.^10^ and Albertsen et al.^11^ primers pairs were tested in silico using RDP ProbeMatch against updated 16S rRNA sequences from all genera available in the current RDP database. As shown in Fig. 1, we found that all Albertsen et al. primer pairs targeting the V1-V3 and V3-V4 and V4 only 16S region showed good performances for bacteria, but had relatively poor performances for archaeal species, that we have shown above to be relevant for wastewater associated communities^12^. On the contrary, Takahashi Pro pair (Pro341F and Pro805R) effectively showed high coverage for both bacteria and archaea, despite a surprisingly low performance for microbes highly relevant for the denitrification cycle, namely anammox bacteria especially of the *Brocadiaceae* family, *Candidatus brocadia* genus. Accordingly, our further efforts were focused on improving the Takahashi et al. primer pair.

### Predicted improvement of coverage on RDP database

**Figure 2:**
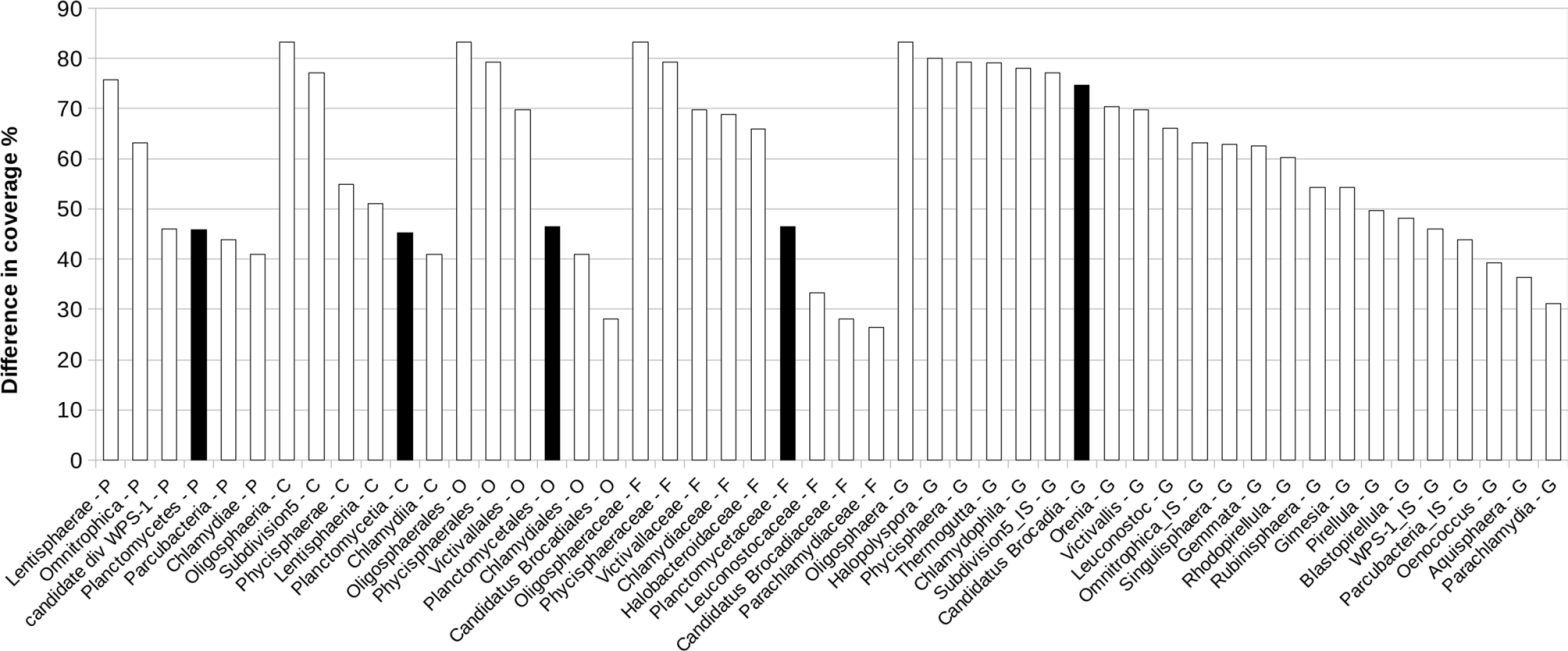
Improvement of taxonomic coverage by the newly optimized primer Pro341FB. The coverage percent value refers to the proportion between the total RDP database sequences annotated with the specific taxonomic rank and those that proved to generate an amplicon using the currently optimized Pro341FB primer and the original Pro341F (white bars), paired with the common reverse primer Pro805R. Only taxonomies with a difference in coverage higher than 25% are shown. The suffixes P, C, O, F and G refers to the ranks phylum, class, order, family and genus, respectively. Black columns mark taxonomic ranks associated with anammox bacteria.

**Figure 3:**
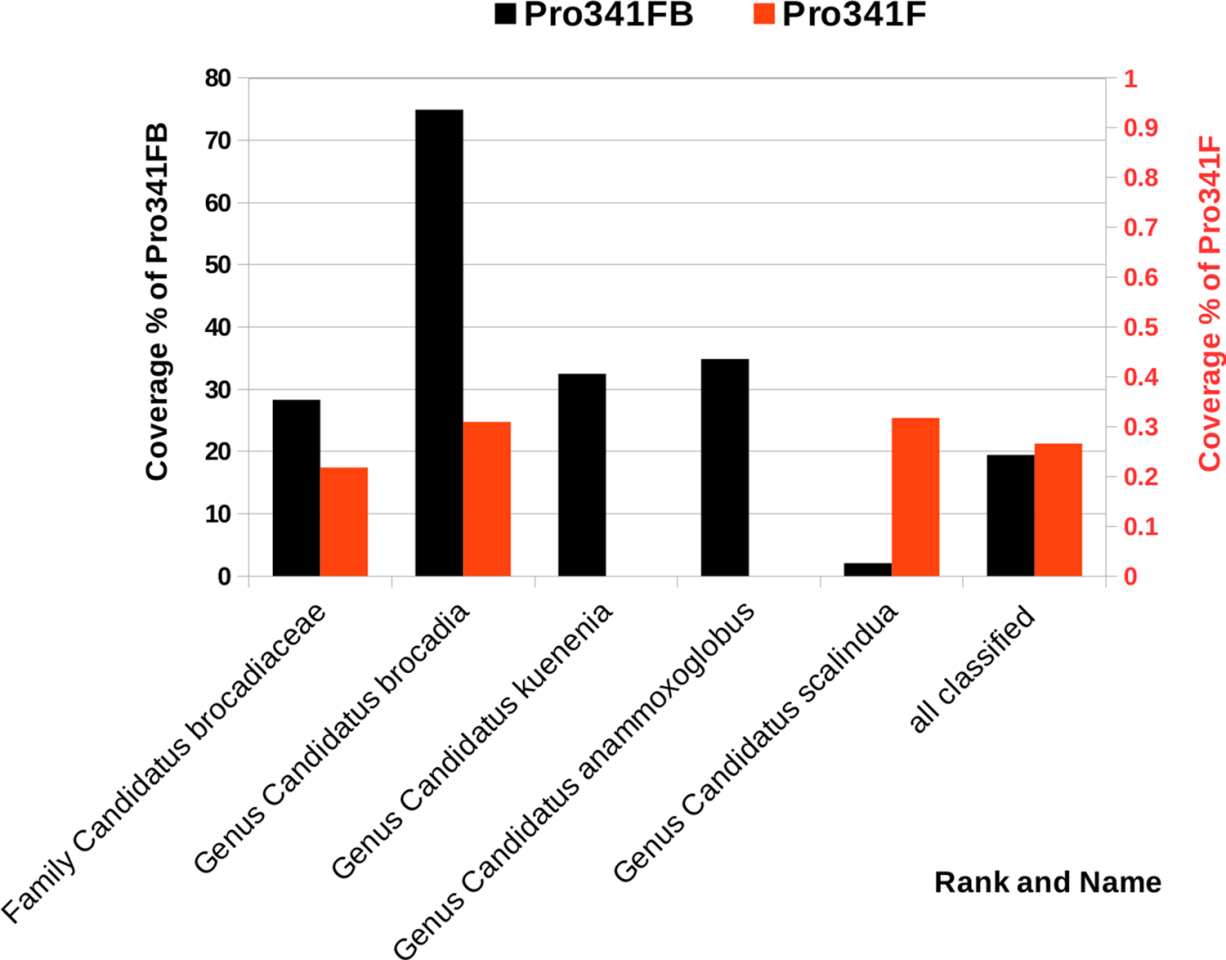
Comparison of the theoretical coverage performance between the new Pro341FB (black, left axis) and the original Pro341F (red, right axis). The *Brocadiaceae* family consists of 5 genera, 4 of which are represented in the figure. A further genus named *Candidatus jettenia* is not present since no high-quality sequence (i.e. > 1200 bp) was present in RDP database.

When specifically matched against *Brocadiales* sequences, we found the possibility of improving the coverage of the Takahashi PRO primer pair by introducing a purine degeneration in the forward primer Pro341F, so that most member of our community of interest was matched. To design this, we extracted from the RDP global dataset all high quality (> 1200 bp) 16S classified as *Brocadiaceae* at the family rank. On this dataset we simulated amplicons formation with RDP probeMatch, systematically imposing degenerations that could accommodate members of this family in the most complete as well as parsimonious way. We ended up with a modified primer, Pro341FB, that was paired with the original reverse primer Pro805R and tested in silico using a mismatch 0 approach and considering the taxonomic coverage as a selection metric. As shown in Fig. 1, the primer pair Pro341FB + Pro805R (TAKB_v3v4) proved a very modest 0.007% coverage increase for archaea with respect to primer Pro341F + Pro805R (TAK_v3v4), while we found a noticeable 1% coverage increase for bacteria. Primer Pro341FB was theoretically able to amplify a total of 59% of the approximately 3.2 million sequences present in the bacteria data bank. In particular, primer Pro341FB was found to target phyla that were completely ignored by the primer Pro341F. As shown in Fig. 2, phyla which received an increase in coverage of more than 25% were found to be *Chlamydiae* (41%), *Lentisphaerae* (76%), *Omnitrophica* (63%), *Parcubacteria* (44%), *candidate division WPS-1* (46%) and, importantly for this study, *Planctomycetes* (46%). Descending the taxonomic tree from phylum Planctomycetes to genera involved in anaerobic ammonium oxidation (anammox) we systematically observed an increase in coverage (class *Planctomycetia* 45%, order *Candidatus Brocadiales* 28%, family *Candidatus Brocadiaceae* 28%, genus *Candidatus Brocadia* 75%). As shown in Fig. 3, all anammox bacteria (genera *Candidatus Brocadia*, *Candidatus Kuenenia*, *Candidatus Anammoxoglobus*, *Candidatus Jettenia* and *Candidatus Scalindua*), that were almost neglected by the original Pro341F primer (red bars, secondary y-axis), resulted, as expected, markedly more covered when the Pro341FB primer was used. Major numerical details on the results of this comparison are available in supplementary materials (Supplementary data S2).

### Testing primer variations by NGS on selected communities

**Figure 4:**
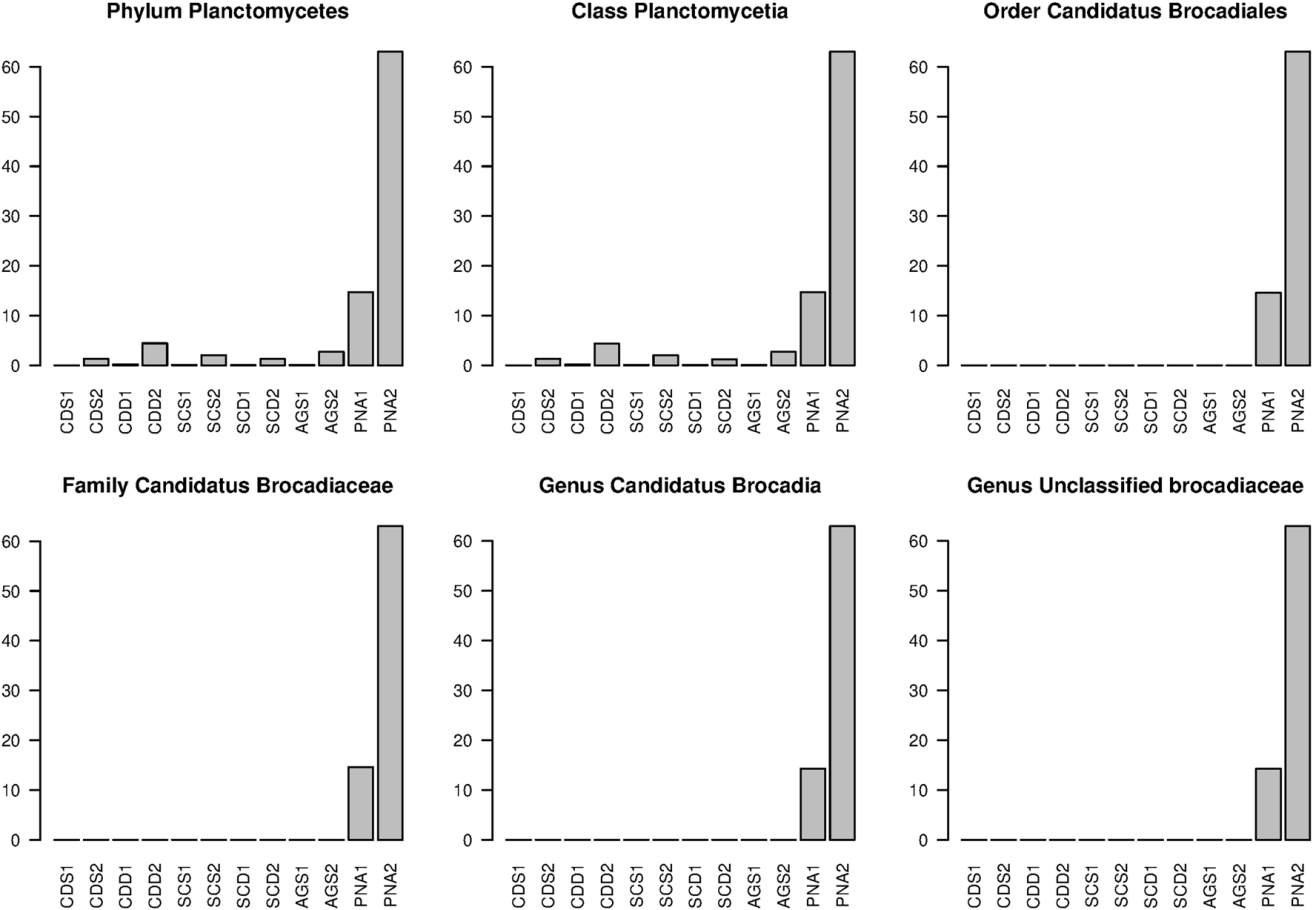
NGS verification of the improvement of coverage percent for members of the *Brocadiaceae* family coverage by the optimized Pro341FB primer. The tested samples (CDS, CDD, SCS, SCD, AGS, PNA, see text for a description) were amplified with Pro341F (suffix 1) or Pro341FB (suffix 2). Samples marked with the suffix 2 are systematically higher in *Brocadia* associated ranks.

In order to verify the increase in performances for anammox communities by our modified forward primer Pro341FB, we collected the microbial community samples from 5 different origins, namely activated sludges from a domestic WWTP plant (SCS), activated sludges from a tannery WWTP (CDS), aerobic granular sludge (AGS), and partial nitrification anammox granular sludge (PNA) from pilot scale reactors fed with domestic wastewater. For the two former plants, samples from their anaerobic digestion reactors were also collected (SCD and CDD, respectively). The samples were collected from bioreactors operated in widely different conditions (suspended vs biofilm and aerobic/anoxic vs anaerobic) and fed with various substrates, in order to allow the validation of the protocol in most of the selective conditions typical for microbial communities in wastewater treatment. The total DNA of all communities was extracted and amplicons were generated using the primer pairs Pro341F + Pro805R or Pro341FB + Pro805R. As shown in Fig. 4, NGS revealed that the percentage of identified phyla was almost the same in all samples but in the PNA, where anammox communities were largely underestimated by Pro341F with respect to Pro341FB. As a confirmation it has been recently reported that anammox species largely dominate the granule population^1^, underlining the underestimation by the original Pro341F primer.

## Discussion

In this work we demonstrated that a minor modification, namely a A/T (IUPAC W) degeneration in the Pro341F forward primer derived from a previously developed primer pair, led to a greatly improved sensitivity for members of the Planctomycetes phylum, that were largely unmatched by the original primer pairs. We further highlighted that this modification has introduced a net improvement, since the performance for phyla other than Planctomycetes was left unaffected by the sequence variation. Accordingly, the optimized forward primer will be of great benefit for the scientific community interested in accurate profiling of prokaryotic populations involved in wastewater treatment. It is important to underline that previous studies focused on the optimization of the primer coverage on eubacteria only. Although eubacteria are largely abundant in wastewater treatment processes, several archaeal species play fundamental roles, especially in methane formation during anaerobic digestion (e.g. for energy recovery) and nitrification, consisting of methanogens and putative ammonia-oxidising archaea (AOA) and belonging, respectively, to the phyla *Euryarchaeota* and *Thaumarchaeota*^12,13^.

The possibility of monitoring the relative abundance of anammox species in wastewater treatment process is of particular interest also due to their low specific growth rates; this makes anammox process the kinetic bottleneck in a biological nutrient removal treatment train and their monitoring is key for processes stability and efficiency. This is a fundamental aspect for currently available anammox-based technologies for the treatment of warm and N-rich wastewaters^14^ and becomes even more crucial when considering the new frontiers of anammox application to colder and more diluted waste streams; this is the case, for example, of the mainstream of domestic wastewater treatment plants where lower operative temperatures would further decrease anammox kinetics^15^. The application of anammox metabolism in the mainstream in fact, would allow the complete uncoupling of carbon and nitrogen removal; this will allow to develop an efficient treatment scheme where carbon is used for energy or matter recovery, ultimately achieving energy autarky and even net energy production for the overall system^16,17^.

For both the study of engineered systems and natural environments, understanding the niche differentiation of the diverse anammox genera would be of primary importance. The widely reported shifts in the dominant anammox populations in different studies^18–20^ supported the notion that anammox bacteria are likely affected by niche differentiation; however, researchers yet failed in an accurate definition^21^. Clearly, the possibility of simultaneous monitoring the evolution of the absolute and relative abundance of different anammox genera in well-defined environments (e.g. lab-scale cultivations) would facilitate this important task.

In conclusion, the improvement in coverage for anammox species brought by our novel primers will be of great benefit, also considering that anammox-based denitrification is considered a novel and promising strategy in the field of wastewater treatment.

## Methods

### In silico screening

The analysis of recurrent genera in waste-water treatment environments was based on a custom perl script that was used to download and parse taxonomic abundance profiles selected and filtered by the online search engine offered by EBI MGnify. Specifically, the database was filtered by imposing ”amplicon” as “experiment type”, and “engineered:wastewater” as “biome”. The search was further filtered by the term “sludge”. The script was designed to download and collect the resulting datasets, extracting counts, statistics and producing informative summary tables.

Primer selection and optimization was performed by a second custom perl script parsing and reorganizing the output of RDP ProbeMatch run on all 16S rRNA sequences available in the current RDP database (release 11 update 5, online since September 2016, 3,356,809 16S rRNA sequences).

All the script used in this work have been deposited and are freely available at https://github.com/matteoramazzotti/wastewater.

### DNA extraction

Samples were collected from different location at the San Colombano (Florence, Italy) and Consorzio CuoioDepur (San Miniato, Pisa, Italy) wastewater treatment plants. Sludges or granular suspensions were centrifuged to recover about 150 mg pelleted material. Pellets were processed using the Qiagen PowerFecal Kit (according to manufacturer instructions) using a FastPrep Beads Beater equipment (ThermoSavant) with a first step of 20″ at 4 m/s followed by a second step of 20″ at 6.5 m/s for initial lysis. DNA concentration and purity was checked by NanoDrop ND-1000 (Thermo Fisher Scientific) and 1.5% agarose (TopVision, Thermo Fisher Scientific) gel electrophoresis (run 30′ at 90 V on a MiniSub Cell GT system, BioRad) stained with SYBR Safe DNA Gel Stain (Invitrogen).

### PCR amplification

For the amplification of the communities for NGS amplicon sequencing the primer pairs Pro341FB/Pro805R or Pro341F/Pro805R were used (see Table 1 for the sequence of the primers). The PCR reaction protocol was based on Thermo Fisher Scientific Platinum SuperFi DNA Polymerase with conditions specified by the NGS service provider, specifically: 98 °C 5′, 25 cycles of 98 °C 30″—56 °C 1′—72 °C 15″, 72 °C 7′. PCR products were checked using 2% gel electrophoresis (see above) and amplicon concentration was estimated by densitometry analysis using quantified bands of the GeneRuler 100 bp DNA ladder (Thermo Fisher Scientific) in a ChemiDoc imaging apparatus and the QuantityOne software (BioRad).

### Sequencing and bioinformatics analysis

PCR amplicons were sent to a commercial sequencing service (BMR Genomics, Padua, Italy) for paired-end 300 bp MiSeq NGS sequencing. For Operational Taxonomic Units (OTU) analysis reads were processed using the MICCA pipeline^22^. Briefly, the obtained demultiplexed reads were assembled with micca mergepair (parameters -l 100 and -d 32), trimmed with micca trim (parameters -W, -R and -c, see Martin 2011) and filtered with micca filter (parameters -m 350 and -e 0.5). Denovo sequence clustering, chimera filtering and taxonomy assignment were performed by micca otu (-m denovo_greedy -d 0.97 -c). An identity of 97% was used to cluster the sequences in OTUs and the longest sequence for each OTU was classified with RDPTools Classifier v 2.12. Further numerical elaborations were performed using the R statistical software.

## Supplementary information

Supplementary file1.

Supplementary file2.
